# Plasma ceramides are associated with MRI-based liver fat content but not with noninvasive scores of liver fibrosis in patients with type 2 diabetes

**DOI:** 10.1186/s12933-023-02049-2

**Published:** 2023-11-08

**Authors:** Damien Denimal, Sarah Béland-Bonenfant, Jean-Paul Pais-de-Barros, Alexia Rouland, Benjamin Bouillet, Laurence Duvillard, Bruno Vergès, Jean-Michel Petit

**Affiliations:** 1 University of Burgundy, INSERM LNC UMR1231, Dijon, F-21000 France; 2 Department of Biochemistry, CHU Dijon Bourgogne, Dijon, F-21079 France; 3 Department of Endocrinology and Diabetology, CHU Dijon Bourgogne, Dijon, F-21000 France; 4 Lipidomic Analytical Platform, University of Burgundy, Dijon, F-21000 France

**Keywords:** Type 2 diabetes, Ceramides, Liver steatosis, Liver fibrosis

## Abstract

**Background:**

There is growing evidence that ceramides play a significant role in the onset and progression of non-alcoholic fatty liver disease (NAFLD), a highly prevalent condition in patients with type 2 diabetes associated with hepatic and cardiovascular events. However, the relationship between plasma ceramide levels and NAFLD severity in type 2 diabetes remains unclear. The main purpose of the present study was to investigate whether circulating levels of ceramides in patients with type 2 diabetes are associated with liver steatosis assessed by the highly accurate magnetic resonance imaging proton density fat fraction (MRI-PDFF). The secondary objective was to assess the relationship between plasma ceramides and noninvasive scores of liver fibrosis.

**Methods:**

In this cross-sectional single-center study, plasma concentrations of 7 ceramides were measured by liquid chromatography-mass spectrometry in 255 patients with type 2 diabetes (GEPSAD cohort). Liver fat content was assessed by MRI-PDFF, and noninvasive scores of liver fibrosis (i.e. Fibrosis-4 index, NAFLD Fibrosis Score, FibroTest® and Fibrotic NASH Index) were calculated. A validation cohort of 80 patients with type 2 diabetes was also studied (LIRA-NAFLD cohort).

**Results:**

Liver steatosis, defined as a liver fat content > 5.56%, was found in 62.4 and 82.5% of individuals with type 2 diabetes in the GEPSAD and LIRA-NAFLD cohorts, respectively. In GEPSAD, MRI-PDFF-measured liver fat content was positively associated with plasma levels of total ceramides (r = 0.232, p = 0.0002), and 18:0, 20:0, 22:0 and 24:0 ceramides in univariate analysis (p ≤ 0.0003 for all). In multivariate analysis, liver fat content remained significantly associated with total ceramides (p = 0.001), 18:0 (p = 0.006), 22:0 (p = 0.0009) and 24:0 ceramides (p = 0.0001) in GEPSAD, independently of age, diabetes duration, body mass index and dyslipidemia. Overall, similar relationship between plasma ceramides and liver fat content was observed in the LIRA-NAFLD validation cohort. No significant association was found between plasma ceramides and noninvasive scores of fibrosis after adjustment for age in both cohorts.

**Conclusions:**

Plasma ceramide levels are associated with liver steatosis in patients with type 2 diabetes, independently of traditional risk factors for NAFLD. The independent association between plasma ceramides and liver steatosis adds new insights regarding the relationship between ceramides and NAFLD in type 2 diabetes.

**Supplementary Information:**

The online version contains supplementary material available at 10.1186/s12933-023-02049-2.

## Background

Non-alcoholic fatty liver disease (NAFLD) is a very common condition in patients with type 2 diabetes, which is associated with an increased risk of adverse hepatic and cardiovascular events [1]. By definition, NAFLD is characterized by the accumulation of triglycerides in the liver, i.e. steatosis [2]. Actually, it encompasses a wide range of conditions, from simple steatosis or non-alcoholic fatty liver (NAFL) to non-alcoholic steatohepatitis (NASH) characterized by necroinflammation with or without fibrosis, which can progress to cirrhosis and even hepatocellular carcinoma [3, 4].

Ceramides are bioactive lipids involved in several mechanisms related to cardiometabolic diseases [5, 6]. In particular, growing evidence suggests that ceramides play a significant role in the onset and progression of NAFLD [7, 8]. In cellular and animal models, ceramides has been found to contribute to key mechanisms of NAFL and NASH, such as hepatic insulin resistance, endoplasmic reticulum stress and mitochondrial dysfunction, which ultimately lead to steatosis, inflammation and fibrosis [7, 8]. Interestingly, decreasing ceramide levels in rodents prevents the development of both liver steatosis and fibrosis [9–11]. In addition, clinical studies have shown that the hepatic ceramide content was associated with liver steatosis and NASH in obese individuals [12, 13].

The interest in circulating ceramide concentrations is currently increasing. Indeed, recent large-scale clinical studies have proposed circulating ceramide levels as biomarkers of cardiovascular events [14–16], including in studies specifically conducted in patients with type 2 diabetes [17]. Clinical data on the relationship between circulating ceramides and NAFLD are still limited and quite heterogeneous, whereas the association between the hepatic ceramide content and NAFLD is better documented [12, 13, 18]. While no association was found between plasma ceramides and liver steatosis or fibrosis in the CERADIAB French cohort of patients with type 2 diabetes [19], other cross-sectional studies have yielded more contrasted results [13, 20, 21]. For instance, some plasma ceramide species were associated with liver steatosis, but others not, in the large Dallas Heart Study, which enrolled nondiabetic individuals [20]. In addition, plasma ceramides were elevated in obese individuals with NASH compared those without NASH, but this association was lost after adjustment for age [21]. Lastly, it was recently reported that plasma ceramide correlates with the extent of liver steatosis in two cohorts containing about 20% of diabetic patients [12, 22].

Drawing a definitive conclusion based on the studies investigating the relationship between plasma ceramides and NAFLD is currently challenging, in particular because liver steatosis has been evaluated using either surrogate biomarkers, or computed tomography- or ultrasound-based methods. In fact, these procedures are consensually recognized to be less accurate than magnetic resonance imaging proton density fat fraction (MRI-PDFF), magnetic resonance spectroscopy (MRS) or liver histology [2, 23]. To the best of our knowledge, only two studies reported data on liver fat content measured by MR-based imaging or histology, but they included no or very few patients with type 2 diabetes, making generalizations to type 2 diabetes uncertain [12, 20].

Clarifying the relationship between plasma ceramides and NAFLD severity in patients with type 2 diabetes would add to our understanding of the interplay between ceramides and NAFLD in this population, especially in the era of antidiabetic drugs such as the GLP-1 receptor agonists that can influence plasma ceramide levels [8]. Therefore, the primary objective of the present study was to investigate whether plasma ceramide levels are related to the liver fat content determined by MRI-PDFF in two independent cohorts composed exclusively of patients with type 2 diabetes. The secondary purpose was to evaluate whether there is any relationship between plasma ceramides and noninvasive scores of liver fibrosis that are Fibrosis-4 index (FIB-4), NAFLD Fibrosis Score (NFS), FibroTest^®^, and Fibrotic NASH Index (FNI).

## Methods

### Study population and design

The present ancillary study included participants enrolled in the single-center GEnetic Polymorphisms, Steatosis and Diabetes (GEPSAD) study. The study complied with the Declaration of Helsinki, and was approved by our local research ethics committee. All patients gave written informed consent before study inclusion. Participants were recruited from October 2007 to September 2010 upon admission to the Department of Diabetology at the Dijon University Hospital (France). The inclusion criteria were: type 2 diabetes, duration of diabetes ≥ 2 years, age ≥ 18 years, and no concurrent acute or chronic disease. Patients were not eligible if they had causes of liver steatosis other than diabetes and overweight [alcohol consumption ≥ 20 g/day for women and ≥ 30 g/day for men, hepatitis B or C virus infection, or use of drugs known to precipitate steatosis (thiazolidinediones, corticosteroids, and immunosuppressants)] or contraindications to MRI (pacemaker, metallic implants, claustrophobia, or body weight > 150 kg).

As a validation cohort, we enrolled participants from the prospective single-center LIRA-NAFLD trial (ClinicalTrials.gov identifier: NCT02721888) [24]. The inclusion criteria were: poorly controlled type 2 diabetes [glycated hemoglobin A1c (HbA1c) > 7.0%), treatment with metformin and/or sulfonylurea and/or insulin. Exclusion criteria were estimated glomerular filtration rate (eGFR) < 30 mL/min/1.73m^2^, pregnancy, alcohol abuse, severe liver impairment defined as aspartate aminotransferase (AST) or alanine aminotransferase (ALT) levels > 3 times the upper limit of normal, treatment with dipeptidyl peptidase-4 inhibitors during the three previous months, or previous treatment with thiazolidinediones or glucagon-like peptide-1 receptor agonist.

For the calculation of sample size, it was assumed that the correlation between plasma ceramide levels and liver fat content is about 0.20 (or an r^2^ of 0.04). Under these assumptions, at least 194 patients were needed to demonstrate a significant effect of plasma ceramide on the liver fat content with an alpha risk of type I error of 5% (bilateral) and a power 1-β of 80%.

During the inclusion visit, patients had a detailed interview, physical examination, liver fat content assessment by MRI-PDFF and fasting blood samples in tubes containing in particular EDTA as preservative for lipidomic analyses. The plasma/serum was immediately separated by centrifugation and analyzed for routine parameters or frozen < -70 °C awaiting lipidomic analyses. Hypertension was defined as systolic blood pressure ≥ 140 mmHg or diastolic blood pressure ≥ 90 mmHg according to American Diabetes Association statement [25], or the use of antihypertensive therapy. Dyslipidemia was defined as triglycerides ≥ 1.70 mmol/L, or high-density lipoprotein (HDL) cholesterol ≤ 1.30 and 1.03 mmol/L for females and males, respectively, or the use of lipid-lowering medications.

### Liver fat content assessment by MRI-PDFF

Liver fat content was assessed using triple-echo MRI-PDFF with a 3.0-Tesla Magnetom Trio whole-body system with total imaging matrix technology (Siemens, Erlangen, Germany), as previously described [26]. Hepatic steatosis was defined as a liver fat content > 5.56% of liver tissue weight (i.e. > 55.6 mg triglycerides/g of liver tissue) [27].

### Noninvasive scores of liver fibrosis

Liver fibrosis was assessed using the noninvasive scores recommended by the European Association for the Study of the Liver (EASL) to rule-out advanced fibrosis in patients with NAFLD/NASH, namely FIB-4, NFS and FibroTest^®^. We also considered FNI as it recently was proved to be accurate [28]. FIB-4, NFS and FNI were calculated according to the original formulas: FIB-4 = (age [years] x AST [IU/L]) / ((platelet count [x10^9^/L]) x (ALT [IU/L])^1/2^); NFS = -1.675 + (0.037 x age [years]) + (0.094 x BMI [kg/m^2^]) + 1.13 + (0.99 x AST/ALT ratio) – (0.013 x platelet count [x10^9^/L]) – (0.66 x albumin [g/dL]); FNI = 𝑒(− 10.33 + 2.54 x ln AST [IU/L] + 3.86 x ln HbA1c [%] − 1.66 x ln HDL-cholesterol [mg/dL]) / (1 + 𝑒(− 10.33 + 2.54 x ln AST [IU/L] + 3.86 x ln HbA1c [%] − 1.66 x ln HDL-cholesterol [mg/dL])) [28–30]. The patented tests (FibroTest^®^ and NashTest^®^) were performed only in the GEPSAD cohort, according to the manufacturer’s instructions (BioPredictive, Paris, France).

### Plasma ceramide measurements by liquid chromatography—tandem mass spectrometry

Plasma levels of the seven main ceramide species with a d18:1 sphingoid backbone were determined as previously described [31]. Briefly, 100 µL plasma were mixed with d18:1/17:0 ceramide (Avanti Polar Lipids, Birmingham, AL, USA), used as internal standard, then extracted with 750 µL of 1:2 chloroform/methanol for 10 min. Chloroform (250 µL) was then added and extraction continued for 10 min more. Water (250 µL) was then added and extraction continued for 10 min more. After centrifugation (9,400 g, 5 min), the organic phase was collected. The aqueous phase was acidified with 8 µL of 3 mol/L of hydrochloric acid, and further extracted with 600 µL of chloroform for 10 min. After centrifugation (9,400 g, 5 min), the two organic phases were combined and washed with 800 µL of the upper phase from a chloroform/methanol/water (96.7:93.3:90) mixture. After centrifugation (9,400 g, 5 min), the organic phase was evaporated under vacuum. Extracts were finally dissolved with 100 µL of 60:30:4.5 chloroform/methanol/water. Three microliters of extract were injected on a 6460 MS/MS system (Agilent Technologies, Les Ulis, France). Separation was achieved on a Poroshell C8 column 2.1 × 100 mm, 2.7-µm (Agilent Technologies) using the following gradient conditions (0.3 mL/min): 1 min at 70% mobile phase B, 70–100% B over 4 min, and 5 min at 100% B. Mobile phases A and B consisted of water and methanol, respectively, both containing 1 mmol/L ammonium formate and 0.2% (v/v) formic acid. Individual ceramide species were identified using multiple-reaction monitoring (Supplementary Table 1). Calibration curves were obtained using external standards (Avanti Polar Lipids) for each ceramide molecular species. The total ceramide concentration was calculated by summing the individual ceramide species. A representative chromatogram is shown in Supplementary Fig.  1.

### Routine laboratory measurements

Routine biochemical parameters [glucose, creatinine, total cholesterol, HDL cholesterol, triglycerides, AST, ALT, gamma-glutamyl transferase (GGT)] were determined on a Dimension Vista platform using dedicated reagents (Siemens, Saint-Denis, France). Low-density lipoprotein (LDL) cholesterol was estimated by the Friedewald formula when serum triglycerides were ≤ 3.8 mmol/L, or was measured using a direct method when serum triglycerides were > 3.8 mmol/L. eGFR was calculated according to the Modification of Diet in Renal Disease equation [32]. The triglyceride-glucose (TyG) index was calculated as defined in Fedchuck et al. [33]. HbA1c was measured by high performance liquid chromatography (Tosoh G8; Tosoh Bioscience, Tokyo, Japan).

### Statistical analysis

Statistical analyses were performed using GraphPad Prism (version 9.1.1). Skewness and kurtosis were calculated for each continuous variable in order to evaluate whether the data distribution matched the Gaussian distribution. For instance, plasma ceramide levels and most of steatosis and fibrosis markers in the present study did not follow a Gaussian distribution, and this was not due to outliers (checked with the robust regression and outlier removal method). Data are reported as medians (interquartile range [IQR]) for non-normally distributed variables or means ± standard deviations (SD) for normally-distributed parameters. A log10 transformation was used for non-normally distributed variables in order to create a Gaussian distribution when necessary for further statistical analyses.

The comparison between two groups of participants was performed using the parametric unpaired Student t test (for normally-distributed variables) and the non-parametric unpaired Mann-Whitney U test (for non-normally distributed variables or when sample size < 30 individuals in a subgroup). Proportions between two groups were compared using the Pearson’s Chi-squared test with Yates’s continuity correction.

For association analysis, univariate Pearson correlation coefficients (r) were determined for continuous variables (using raw data for normally-distributed variables and log10-transformed data for non-normally distributed variables). Point biserial correlation was used to assess the correlations between continuous variables and categorical variables. A Bonferroni correction procedure was applied for controlling false positives in multiple testing, by dividing the desired alpha-level (i.e. 0.05) by the number of comparisons. Multivariate analyses were performed by multivariate linear or logistic regression for continuous and categorical dependent variables, respectively.

A two-tailed probability level of 0.05 was considered statistically significant for all statistical analyses.

## Results

### Participant characteristics

On the 298 participants with type 2 diabetes enrolled in the GEPSAD cohort, 29 and 14 individuals were excluded from the present study due to the lack of MRI-PDFF and ceramide measurements, respectively. This resulted in a total of 255 participants, whose characteristics are presented in Table 1. Participants exhibited typical features of type 2 diabetes, such as increased body mass index (> 25 and > 30 kg/m^2^ in 95.3 and 70.6% of patients, respectively), elevated triglyceride levels (≥ 1.70 mmol/L in 50.2% of patients), low plasma HDL-cholesterol concentrations (≤ 1.30 mmol/L in 72.9% of females and ≤ 1.03 mmol/L in 62.4% of males), and elevated TyG index (> 8.38 in 94.5% of participants). AST and ALT ≥ 30 IU/L were found in 64 (25.1%) and 143 (56.1%) participants, respectively.

We also studied another cohort of patients with type 2 diabetes as a validation cohort. The 80 participants were initially enrolled in the LIRA-NAFLD trial, and their main characteristics are presented in Supplementary Table  2.

**Table 1:** Patient characteristics in GEPSAD

Characteristic	All	LFC ≤ 5.56%	LFC > 5.56%
No. participants	255	96	159
**Clinical characteristics**			
Age, years	60.3 ± 10.1	62.5 ± 9.8	59.0 ± 9.9 **
Gender, % female (n)	51.0 (130)	50.0 (48)	51.6 (82)
Body mass index, kg/m^2^	34.2 ± 6.4	32.1 ± 6.4	35.5 ± 6.1 ***
≥ 30 kg/m^2^, % (n)	71.8 (183)	55.2 (53)	81.8 (130) ***
Diabetes duration, years	13.4 ± 10.0	16.0 ± 10.3	11.8 ± 9.6 **
Tobacco (current or former), % (n)	54.5 (139)	50.0 (48)	57.2 (91)
Hypertension, % (n)	85.9 (219)	92.7 (89)	81.8 (130) *
Retinopathy, % (n)	30.2 (77)	38.5 (37)	25.2 (40) *
Diabetes medications			
Insulin, % (n)	60.8 (155)	69.8 (67)	55.4 (88) *
Metformin, % (n)	56.5 (144)	40.6 (39)	66.0 (105) ***
Sulfonylureas, % (n)	51.0 (130)	49.0 (47)	52.2 (83)
GLP-1 receptor agonists, % (n)	5.5 (14)	3.1 (3)	6.9 (11)
Glucosidase inhibitors, % (n)	2.7 (7)	2.1 (2)	3.1 (5)
Lipid-lowering agents			
Statins, % (n)	52.2 (133)	66.7 (64)	43.4 (69) ***
Fibrates, % (n)	15.3 (39)	4.2 (4)	22.0 (35) ***
Ezetimibe, % (n)	5.1 (13)	5.3 (5)	5.0 (8)
**Routine blood markers**			
HbA1c, %	8.6 ± 1.8	8.5 ± 1.9	8.7 ± 1.7
HbA1c, mmol/mol	71 ± 15	69 ± 15	72 ± 16
Fasting plasma glucose, mmol/L	8.9 [7.1–11.1]	8.3 [6.5–11.1]	9.1 [7.2–11.1]
eGFR, mL/min/1.73m^2^	99 [77–130]	84 [66–115]	111 [86–142] ***
Total cholesterol, mmol/L	4.78 ± 1.15	4.59 ± 1.21	4.90 ± 1.10
LDL-cholesterol, mmol/L	2.80 ± 0.96	2.72 ± 1.02	2.85 ± 0.93
HDL-cholesterol, mmol/L	1.10 ± 0.26	1.13 ± 0.30	1.08 ± 0.24
Triglycerides, mmol/L	1.70 [1.21–2.67]	1.36 [1.05–2.02]	2.04 [1.42–2.88] ***
TyG index	9.48 ± 0.75	9.22 ± 0.70	9.64 ± 0.74 ***
AST, IU/L	20.0 [13.8–29.0]	19.5 [12.0–24.0]	22.0 [15.0–32.0] **
ALT, IU/L	32.0 [24.8–47.0]	26.0 [21.0–32.0]	38.0 [59.0–53.0] ***
GGT, IU/L	41.0 [26.0–72.0]	33.5 [22.3–55.5]	46.0 [32.0–78.0] **

Data are means ± SD (for normally-distributed variables), medians [IQR] (for non-normally distributed variables) or percentages, as appropriate

Abbreviations: AST, aspartate aminotransferase; ALT, alanine aminotransferase; GGT, gamma-glutamyl transferase; eGFR, estimated glomerular filtration rate; GLP-1, glucagon-like peptide-1; HDL, high-density lipoprotein; LDL, low-density lipoprotein; LFC, liver fat content; TyG, triglyceride-glucose index

LFC ≤ 5.56% vs. LFC > 5.56%: *p < 0.05, **p < 0.01, and ***p < 0.001

### Liver fat content assessment by MRI-PDFF

Liver steatosis, defined as a liver fat content > 5.56% using MRI-PDFF, was found in 159 (62.4%) and 66 (82.5%) participants with type 2 diabetes in GEPSAD and LIRA-NAFLD, respectively. As shown in Table 1 and Supplementary Tables  2, individuals with liver steatosis had higher body mass index (and plasma ALT than individuals without liver steatosis in both cohorts, while levels of HbA1c were similar (p = 0.37 and 0.85 in GEPSAD and LIRA-NAFLD, respectively).

### Noninvasive scores of liver fibrosis

Noninvasive scores of liver fibrosis are shown in Table 2 and Supplementary Table  4 for GEPSAD and LIRA-NAFLD, respectively. About three quarters of participants in both cohorts were considered at a low risk of having advanced fibrosis based on the FIB-4 threshold of 1.30 recommended in the European and US guidelines [34, 35]. This increased to 90% in GEPSAD according to the FibroTest^®^ score (< 0.48).

**Table 2:** Liver fat content and noninvasive scores of liver fibrosis in GEPSAD

Characteristic	All	LFC ≤ 5.56%	LFC > 5.56%
No. subjects	255	96	159
**Liver fat content using MRI-PDFF**			
Liver fat content (%)	8.96 [3.32–16.6]	2.70 [1.58–4.11]	13.9 [9.42–21.2]***
> 5.56%, % (n)	62.4 (159)	0.0 (0)	100 (159)***
**Noninvasive scores of fibrosis**			
FIB-4	0.875 [0.594–1.345]	0.918 [0.603–1.359]	0.818 [0.576–1.344]
< 1.30, % (n)	72.4 (181)	74.5 (70)	71.2 (111)
≥ 2.67, % (n)	3.6 (9)	4.3 (4)	3.2 (5)
NFS	-0.163 ± 1.220	-0.078 [-0.869-0.669]	0.004 [-0.890-0.704]
< -1.455, % (n)	11.7 (26)	12.8 (10)	11.1 (16)
≥ 0.676, % (n)	25.2 (56)	24.4 (19)	25.7 (37)
FibroTest^®^ score	0.187 [0.081–0.316]	0.191 [0.105–0.280]	0.180 [0.078–0.327]
≤ 0.48, % (n)	90.1 (219)	93.3 (84)	88.2 (135)
> 0.58, % (n)	6.2 (15)	4.4 (4)	7.2 (11)
FNI	0.394 ± 0.282	0.247 [0.119–0.471]	0.444 [0.194–0.672]**
< 0.10, % (n)	18.5 (46)	23.7 (22)	15.4 (24)
≥ 0.33, % (n)	51.4 (128)	38.7 (36)	59.0 (92)**
**Inflammation score**			
NashTest^®^ score	0.491 ± 0.182	0.440 ± 0.155	0.521 ± 0.190***
0.25, % (n)	29.0 (70)	34.1 (31)	25.0 (39)
0.75, % (n)	25.3 (61)	9.9 (9)	33.3 (52)***

Data are means ± SD (for normally-distributed variables), medians [IQR] (for non-normally distributed variables) or percentages, as appropriate. FIB-4, NFS, FibroTest^®^ and FNI were determined in 250, 222, 243 and 249 patients, respectively, in particular due to the lack of platelet count or albumin values in certain individuals

Abbreviations: FIB-4, fibrosis-4 index; FNI, fibrotic NASH index; LFC, liver fat content; MRI-PDFF, magnetic resonance imaging proton density fat fraction; NFS, NAFLD fibrosis score

LFC ≤ 5.56% vs. LFC > 5.56%: **p < 0.01, and ***p < 0.001

### Plasma ceramides and liver steatosis

The main purpose of the study was to investigate the relationship between plasma ceramide levels and liver fat content determined by MRI-PDFF in patients with type 2 diabetes. In GEPSAD, participants with liver steatosis according to MRI-PDFF had higher circulating concentrations of total ceramides (median [IQR], 5.53 [4.31–7.52] vs. 5.07 [3.86–6.46] µmol/L, p = 0.02), 18:0 ceramide (0.126 [0.088–0.164] vs. 0.071 [0.056–0.098] µmol/L, p = 0.009), 20:0 ceramide (0.084 [0.058–0.107] vs. 0.071 [0.056–0.098] µmol/L, p = 0.05), 22:0 ceramide (0.595 [0.446–0.809] vs. 0.544 [0.361–0.706] µmol/L, p = 0.01) and 24:0 ceramide (3.12 [2.25–4.27] vs. 2.78 [1.95–3.64] µmol/L, p = 0.01) compared to the patients without liver steatosis.

**Fig. 1:**
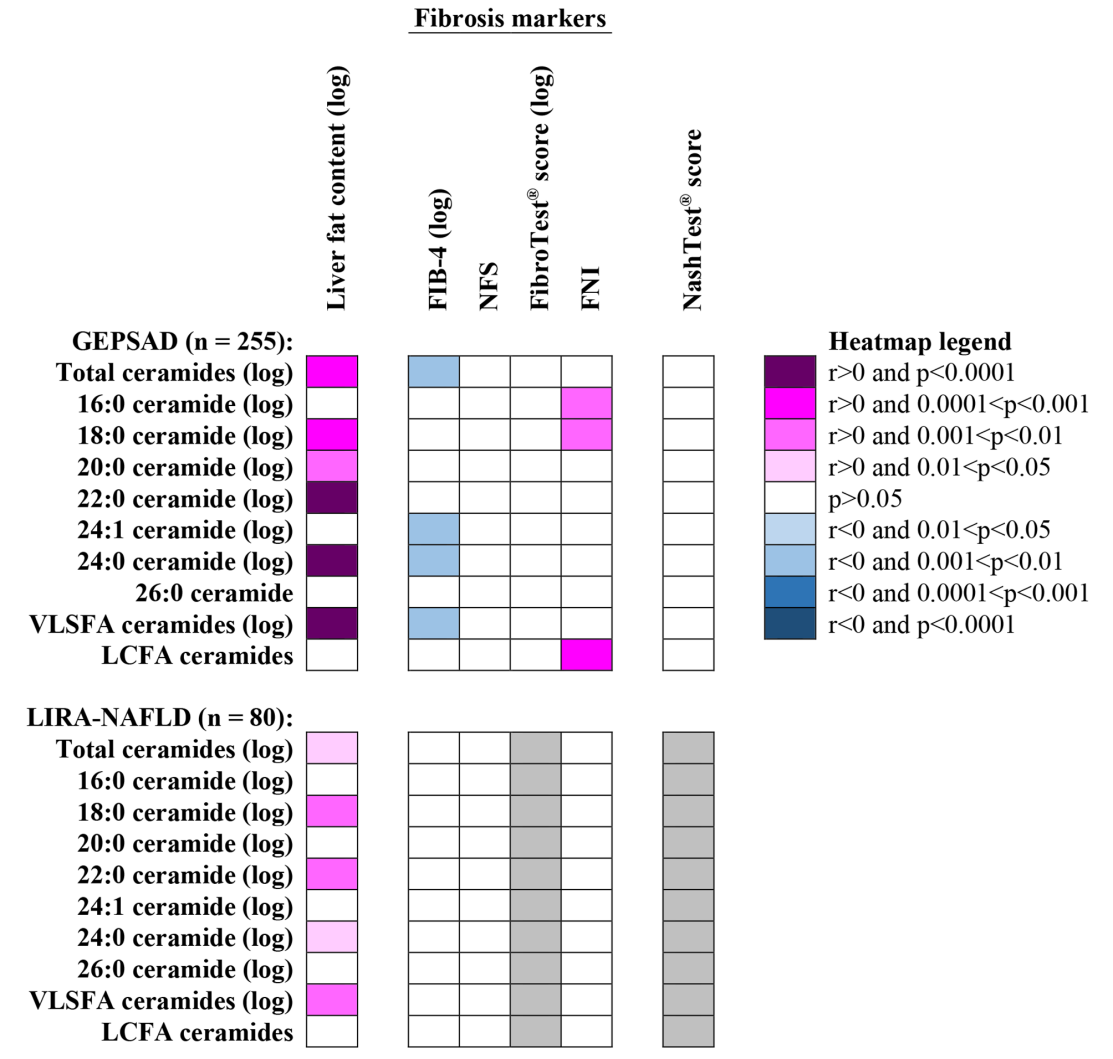
Univariate correlations between plasma ceramide concentrations and hepatic markers of NAFLD in GEPSAD and LIRA-NAFLD (shown as a heatmap). Only correlations remaining statistically significant after Bonferroni correction were reported in the heatmap (i.e. when p < 0.0063). Liver fat content corresponds to the value determined by MRI-PDFF. FibroTest^®^ and NashTest^®^ were not performed in LIRA-NAFLD (grey cells). Abbreviations: FIB-4, fibrosis-4 index; FNI, fibrotic NASH index; LCFA, long-chain fatty acid; NFS, NAFLD fibrosis score; VLSFA, very long-chain saturated fatty acid

Figure 1 shows the univariate correlations between plasma ceramides and liver fat content assessed by MRI-PDFF. After Bonferroni adjustment, the liver fat content remained positively associated with plasma total ceramides (r = 0.232, p = 0.0002), 18:0 (r = 0.241, p = 0.0001), 20:0 (r = 0.183, p = 0.0003), 22:0 (r = 0.255, p < 0.0001) and 24:0 ceramides (r = 0.256, p < 0.0001) in GEPSAD. We were interested in grouping together ceramide species with very long-chain saturated fatty acids (VLSFA), i.e. with at least 20 carbon atoms, because they are linked to health outcomes in epidemiological studies in a different manner than the ceramides with long-chain fatty acids (LCFA, i.e. the 16:0 and 18:0 ceramides) [36]. In GEPSAD, the liver fat content correlated with the plasma concentration of ceramides containing VLSFA (r = 0.264, p < 0.0001), but not with ceramides containing LCFA (r = 0.096, p = 0.127).

Not surprisingly, liver fat content correlated with age, diabetes duration, body mass index and dyslipidemia in univariate analysis (data not shown). Using a multivariate linear regression model including all these variables (Table 3), the liver fat content measured by MRI-PDFF remained independently associated with plasma total ceramides in GEPSAD [standardized β (95% confidence intervals (CI)), 0.201 (0.082–0.320), p = 0.001]. Using a multivariate logistic regression approach, plasma levels of total ceramides were independently linked to the presence of liver steatosis, i.e. with liver fat content > 5.56% [odds-ratio (OR) (95% CI), 8.2 (1.6–46), p = 0.014)] (Supplementary Table  3).

**Table 3:** Multivariate linear regression analysis for MRI-PDFF-based liver fat content (log)

	GEPSAD (n = 255)		LIRA-NAFLD (n = 80)	
	Standardized β [95% CI]	P-value	Standardized β [95% CI]	P-value
**Total ceramides (log)**	**0.201 [0.082 0.320]**	**0.001**	**0.252 [0.029–0.475]**	**0.032**
16:0 ceramide (log)	0.001 [-0.119 0.122]	0.98	0.022 [-0.067–0.109]	0.68
**18:0 ceramide (log)**	**0.170 [0.050 0.290]**	**0.006**	**0.293 [0.064–0.522]**	**0.009**
**20:0 ceramide (log)**	**0.138 [0.019–0.257]**	**0.02**	0.141 [-0.067–0.275]	0.19
**22:0 ceramide (log)**	**0.203 [0.084 0.322]**	**0.0009**	**0.287 [0.052–0.522]**	**0.010**
24:1 **ceramide** (log)	0.102 [-0.019–0.223]	0.10	0.136 [-0.090–0.362]	0.26
**24:0 ceramide (log)**	**0.235 [0.117–0.353]**	**0.0001**	**0.263 [0.031–0.495]**	**0.024**
26:0 ceramide	-0.059 [-0.178–0.061]	0.33	0.021 [-0.145–0.187]	0.47
**VLSFA ceramides (log)**	**0.237 [0.119**–**0.355]**	**0.0001**	**0.245 [0.012–0.478]**	**0.037**
LCFA ceramides	0.059 [-0.060–0.178]	0.32	-0.023 [-0.112–0.066]	0.69

The model is adjusted for age, diabetes duration, body mass index and dyslipidemia. Bolded: p < 0.05

Abbreviations: CI, confidence intervals; LCFA, long-chain fatty acid; VLSFA, very long-chain saturated fatty acid

Regarding ceramide molecular species in multivariate linear regression analysis (Table 3), the liver fat content measured by MRI-PDFF was independently associated with the plasma concentrations of 18:0 ceramide [standardized β (95% CI), 0.170 (0.050–0.290), p = 0.006], 20:0 ceramide [0.138 (0.019–0.257), p = 0.02], 22:0 ceramide [0.203 (0.084–0.322), p = 0.0009] and 24:0 ceramide [0.235 (0.117–0.353), p = 0.0001] in GEPSAD. The plasma concentration of ceramides with VLSFA was independently linked to the liver fat content, while the plasma concentration of ceramides with LCFA were not.

We aimed to confirm these results using the LIRA-NAFLD validation cohort, composed of 80 patients with type 2 diabetes. Similarly to GEPSAD, the liver fat content measured by MRI-PDFF in LIRA-NAFLD was also positively associated with the plasma concentration of total ceramides (r = 0.244, p = 0.029), 18:0 ceramide (r = 0.293, p = 0.008), 22:0 ceramide (r = 0.320, p = 0.003), 24:0 ceramide (r = 0.253, p = 0.016) and ceramides containing VLSFA (r = 0.280, p = 0.012) in univariate analysis (Fig. 1). In multivariate analysis, liver fat content remained independently associated with plasma total ceramides, 18:0 ceramide, 22:0 ceramide, 24:0 ceramide, and with ceramides containing VLSFA (Table 3).

### Plasma ceramides and noninvasive scores of liver fibrosis

The secondary objective of the study was to investigate the relationship between plasma ceramides and noninvasive scores of liver fibrosis. The participants were divided into two groups according to the thresholds of noninvasive scores usually recommended to rule-out significant fibrosis (27,31). In GEPSAD, the circulating concentrations of total ceramides were higher in participants with FIB-4 < 1.30 than in those with FIB-4 ≥ 1.30 (median [IQR], 5.53 [4.36–7.42] vs. 4.95 [3.77–6.41] µmol/L, p = 0.04), and were similar between participants with NFS < or ≥ -1.455 (6.04 [4.58–7.47] vs. 5.36 [4.17–7.10] µmol/L, p = 0.28), FibroTest^®^ score ≤ or > 0.48 (5.40 [4.18–7.24] vs. 4.91 [4.14–7.08] µmol/L, p = 0.38) and FNI < or ≥ 0.10 (5.55 [4.40–6.59] vs. 5.33 [4.17–7.28] µmol/L, p = 0.83).

As shown in Fig. 1, few correlations between plasma ceramides and fibrosis scores remained significant after Bonferroni correction in univariate regression in both cohorts. Age was strongly associated with all scores of fibrosis in both cohorts (p < 0.0001 for all). Using multivariate linear regression analysis, the plasma concentration of total ceramides was not significantly associated with FIB-4 [standardized β (95% CI), -0.109 (-0.225–0.006), p = 0.06], NFS [-0.077 (-0.203–0.049), p = 0.23], FibroTest^®^ [0.063 (-0.051–0.176), p = 0.28] and FNI [0.004 (-0.122–0.131), p = 0.95] in GEPSAD after adjustment for age. Similar results were obtained using a multivariate logistic regression approach by considering thresholds usually recommended to rule-out significant fibrosis (Table 4) [28, 34]. Since preliminary evidence suggests that FIB-4 outperforms NFS and FibroTest^®^ to rule in advanced fibrosis in individuals with type 2 diabetes [34, 37], we also assessed the relationship between plasma ceramides and FIB-4 at the rule-in threshold for advanced fibrosis using multivariate analysis. Thus, no plasma ceramide level was associated with a FIB-4 ≥ 2.67 in GEPSAD after adjustment for age (e.g., estimate [95% CI], OR = 0.024 [3 × 10^− 4^–1.70], p = 0.09 for total ceramides).

**Table 4:** Multivariate logistic regression analysis for noninvasive scores of fibrosis

	GEPSAD		LIRA-NAFLD	
	Odds-ratio [95% CI]	P-value	Odds-ratio [95% CI]	P-value
**FIB-4 ≥ 1.30**				
Total ceramides (log)	0.48 [0.08–2.7]	0.41	0.18 [8 × 10^− 3^–2.8]	0.24
16:0 ceramide (log)	**4655 [1.5–2 × 10** ^**7**^ **]**	**0.04**	10 [7 × 10^− 3^–8 × 10^4^]	0.54
18:0 ceramide (log)	4.1 [0.0–9 × 10^5^]	0.87	0.72 [0.27–2.0]	0.52
20:0 ceramide (log)	1.2 [0.2–6.1]	0.87	0.65 [0.14–3.1]	0.57
22:0 ceramide (log)	1.1 [0.3–5.0]	0.15	1.4 [0.12–15]	0.78
24:1 ceramide (log)	0.46 [0.09–2.2]	0.34	0.50 [0.05–4.5]	0.53
24:0 ceramide (log)	0.42 [0.09–1.9]	0.26	0.13 [8 × 10^− 3^–1.7]	0.13
26:0 ceramide	**2.6 [1.1–6.4]**	**0.04**	0.14 [5 × 10^− 3^–3.0]	0.22
VLSFA ceramides (log)	0.48 [0.10–2.3]	0.36	0.18 [0.01–2.5]	0.22
LCFA ceramides	4.4 [0.63–30.6]	0.13	1.0 [0.98–1.1]	0.27
**NFS ≥ -1.455**				
Total ceramides (log)	0.61 [0.05–6.9]	0.40	0.11 [3 × 10^− 4^–17]	0.79
16:0 ceramide (log)	1 × 10^− 4^ [4 × 10^− 9^–6]	0.10	1 × 10^7^ [8.5–5 × 10^16^]	0.06
18:0 ceramide (log)	5 × 10^− 6^ [1 × 10^− 14^–2996]	0.22	0.78 [0.13–6.6]	0.79
20:0 ceramide (log)	0.16 [0.01–1.7]	0.14	0.65 [0.03–23]	0.79
22:0 ceramide (log)	0.43 [0.05–3.2]	0.42	0.27 [5 × 10^− 3^–11]	0.50
24:1 ceramide (log)	1.3 [0.1–12]	0.82	0.06 [5 × 10^− 4^–5.9]	0.23
24:0 ceramide (log)	0.7 [0.08–6.3]	0.76	0.24 [2 × 10^− 3^–17]	0.53
26:0 ceramide	3 × 10^− 5^ [7 × 10^− 12^–227]	0.19	1.26 [5 × 10^− 3^–292]	0.93
VLSFA ceramides (log)	0.62 [0.06–5.7]	0.67	0.19 [1 × 10^− 3^–17]	0.49
LCFA ceramides	0.13 [0.01–1.7]	0.11	1.0 [0.95–1.1]	0.65
**FibroTest® > 0.48**				
Total ceramides (log)	0.94 [0.06–15]	0.97	N.A.	N.A.
16:0 ceramide (log)	6 × 10^− 6^ [1 × 10^− 12^–4.4]	0.10	N.A.	N.A.
18:0 ceramide (log)	0.01 [7 × 10^− 14^–2 × 10^8^]	0.72	N.A.	N.A.
20:0 ceramide (log)	0.73 [0.05–11]	0.81	N.A.	N.A.
22:0 ceramide (log)	0.58 [0.06–5.9]	0.64	N.A.	N.A.
24:1 ceramide (log)	2.4 [0.2–30]	0.48	N.A.	N.A.
24:0 ceramide (log)	0.80 [0.07–9.0]	0.85	N.A.	N.A.
26:0 ceramide	**4 × 10** ^**− 11**^ **[1 × 10** ^**− 21**^ **− 0.05]**	**0.04**	N.A.	N.A.
VLSFA ceramides (log)	0.80 [0.07–9.7]	0.86	N.A.	N.A.
LCFA ceramides	0.10 [2 × 10^− 3^–2.6]	0.19	N.A.	N.A.
**FNI ≥ 0.10**				
Total ceramides (log)	1.03 [0.16–6.8]	0.97	17 [0.65–612]	0.09
16:0 ceramide (log)	1968 [0.2–4 × 10^7^]	0.11	1.2 [4 × 10^− 4^–4270]	0.96
18:0 ceramide (log)	**2 × 10** ^**11**^ **[2 × 10** ^**3**^ **− 2 × 10** ^**20**^ **]**	**0.009**	1.5 [0.41–4.7]	0.50
20:0 ceramide (log)	4.2 [0.7–25]	0.11	2.0 [0.28–12]	0.45
22:0 ceramide (log)	2.2 [0.4–11]	0.34	15 [0.76–475]	0.10
24:1 ceramide (log)	1.2 [0.2–6.6]	0.87	9.1 [0.74–130]	0.08
24:0 ceramide (log)	0.60 [0.1–3.1]	0.54	9.9 [0.49–250]	0.14
26:0 ceramide	114 [0.0–9 × 10^7^]	0.48	1.00 [0.90–1.08]	0.99
VLSFA ceramides (log)	0.77 [0.14–4.2]	0.76	14 [0.60–418]	0.11
LCFA ceramides	**13 [1.2–168]**	**0.04**	1.01 [0.97–1.07]	0.58

The model is adjusted for age. The thresholds are those usually recommended to rule-out significant fibrosis [28, 34]. FibroTest^®^ was not performed in LIRA-NAFLD. Bolded: p < 0.05

Abbreviations: CI, confidence intervals; FIB-4, fibrosis-4 index; FNI, fibrotic NASH index; LCFA, long-chain fatty acid; N.A., not applicable; NFS, NAFLD fibrosis score; VLSFA, very long-chain saturated fatty acid

In the LIRA-NAFLD validation cohort, the plasma concentration of total ceramides was also not associated with the FIB-4 [standardized β (95% CI), -0.092 (-0.276–0.093), p = 0.32], NFS [1.5 × 10^− 5^ (-3.4 × 10^− 4^–3.7 × 10^− 4^), p = 0.93], and FNI [1.0 × 10^− 5^ (-1.3 × 10^− 5^–3.3 × 10^− 5^), p = 0.41] scores after adjustment for age. Overall, the multivariate logistic regression approach yielded similar findings than in GEPSAD using the rule-out thresholds for advanced fibrosis (Table 4).

## Discussion

Here, our key finding was that plasma ceramide levels correlate positively with the degree of liver steatosis, namely with the liver fat content assessed by MRI-PDFF expressed as percentage, in two independent cohorts including overall 335 individuals with type 2 diabetes. The association between plasma ceramides and liver fat content was independent of traditional risk factors for NAFL. Interestingly, an Australian study has also shown that plasma ceramide levels are linked to the extent of liver steatosis using histology, which is another reference method [12]. But, this study was conducted in obese patients of which only 22% had type 2 diabetes, making generalization to all patients with type 2 diabetes uncertain. Our results suggest that this link also exists in patients with type 2 diabetes. Another clinical study, conducted in 149 patients with type 2 diabetes, found a positive association between plasma total ceramides and liver steatosis [19], but using surrogate biomarkers of steatosis (i.e. SteatoTest^®^ and fatty liver index) that are extensively recognized as being less accurate than MR-based imaging methods [34]. In contrast to our results, Apostolopoulou et al. found that the circulating levels of total ceramides were not significantly higher in obese individuals with biopsy-proven liver steatosis than in those without liver steatosis, but this study included only 7 individuals in each group [13].

Regarding ceramide molecular species, we found an independent association between liver fat content and the plasma levels of the 18:0, 20:0, 22:0 and 24:0 ceramide species. Similar results were also observed in the Australian study mentioned above, which was conducted in obese participants including 22% with type 2 diabetes [12]. A US study found that plasma 18:0 ceramide was independently associated with the extent of steatosis, but the 20:0, 22:0 and 24:0 species were not [22]. We hypothesized that the difference with our results regarding these ceramides with VLSFA could be due to the fact that liver steatosis was assessed by non-contrast computed tomography, which is a less accurate method than MRI-PDFF, and perhaps also because of a particular study population of Asian individuals living in USA. In contrast to our results, the Dallas Heart Study found that the plasma level of 18:0 ceramide was not associated with MRS-measured liver fat content in a large population without diabetes [20]. However, the link between plasma ceramides and liver steatosis can be quite different in patients with type 2 diabetes since the plasma concentration of 18:0 ceramide is largely increased in this population [38–40]. Overall, we found an independent association in our two cohorts between liver fat content and plasma ceramides with VLSFA. Interestingly, decreasing specifically these ceramide species in mice reduces liver steatosis [41].

Our secondary aim was to investigate the link between plasma ceramides and noninvasive scores of liver fibrosis, since cellular and animal studies suggest that ceramides could play a role in hepatic fibrogenesis [9]. Overall, we found no association between plasma ceramide levels and noninvasive scores of liver fibrosis in both cohorts. In particular, this was confirmed using the FIB-4, FibroTest^®^ and NFS scores, which are all recognized as useful for ruling-out advanced fibrosis in guidelines [2, 34]. Other clinical studies on ceramides and liver fibrosis are sparse, but a lack of relationship between plasma ceramides and FibroTest^®^ has been already observed in two French cohorts of patients with type 2 diabetes with fewer participants than in our study [19]. In a context other than type 2 diabetes, it has been demonstrated that plasma ceramide concentrations are not linked to severe fibrosis stage assessed by liver biopsy in individuals with chronic hepatitis C [42]. However, it has been shown that plasma ceramides decreased with the severity of liver cirrhosis, but in a cirrhotic population including only 2% patients with NAFLD [43]. Overall, our results suggest that plasma ceramides are not useful as clinical biomarkers of liver fibrosis in patients with type 2 diabetes.

The main strength of our study is that it reports on the relationship between plasma ceramides and the extent of liver steatosis using the accurate MRI-PDFF procedure, and also that it focuses specifically on a population with type 2 diabetes. MRI-PDFF is recognized by the EASL to be the most accurate non-invasive method for detecting and quantifying steatosis [34]. However, our study has limitations that are worth discussing. Firstly, our two cohorts are relatively small, which can diminish the power of our correlations. Secondly, medications for management of diabetes and dyslipidemia may change ceramide metabolism and subsequently the relationship between plasma ceramides and liver outcomes. Although the effects of lipid-lowering drugs on ceramides have not been studied extensively, it has been reported that statins reduce plasma ceramide levels, unlike ezetimibe [44, 45]. However, introducing the use of statins in our multivariate analysis did not significantly change the association between plasma ceramide levels and liver fat content (data not shown), suggesting that statin use was not a confounding factor in our study. Thirdly, the lack of liver biopsy or magnetic resonance elastography to diagnose fibrosis should be mentioned as a limitation for accurately investigating the relationship between fibrosis and plasma ceramides. However, the noninvasive scores of liver fibrosis used in the present study are well-validated, and the guidelines consider that they are sufficiently accurate to rule-out advanced fibrosis in individuals with NAFLD [2, 34]. Lastly, the GEPSAD study has a cross-sectional design, and as such, demonstrates correlation between plasma ceramides and liver fat content, but not causation.

## Conclusions

Plasma levels of ceramides are associated with the degree of liver steatosis in patients with type 2 diabetes, but not with noninvasive scores of liver fibrosis. Our results help to clarify the relationship between plasma ceramides and NAFLD severity, and therefore add to the knowledge of the interplay between ceramides and NAFLD in type 2 diabetes. However, longitudinal studies are needed to determine whether plasma ceramide levels can predict the progression to advanced stages of NAFLD in patients with type 2 diabetes.

### Electronic supplementary material

Below is the link to the electronic supplementary material.

**Supplementary Table 1**. Quantified ceramide species and conditions for mass spectrometry acquisition.

**Supplementary Table 2**. Patient characteristics in LIRA-NAFLD.

**Supplementary Table 3**. Multivariate logistic regression analysis for liver fat content > 5.56%.

**Supplementary Table 4**. Liver fat content and noninvasive scores of liver fibrosis in LIRA-NAFLD.

**Supplementary Fig. 1**. A representative chromatogram of external and internal standards.

## Data Availability

The datasets supporting the conclusions of this article are available from the corresponding author on reasonable request.

## Abbreviations

ALT: alanine aminotransferase
AST: aspartate aminotransferase
CI: confidence interval
EASL: European Association for the Study of the Liver
EDTA: ethylenediaminetetraacetic acid
eGFR: estimated glomerular filtration rate
FIB-4: fibrosis-4 index
FNI: fibrotic NASH index
GEPSAD: genetic polymorphisms, steatosis and diabetes
GGT: gamma-glutamyl transferase
GLP-1: glucagon-like peptide-1
HDL: high-density lipoprotein
IQR: interquartile range
LCFA: long-chain fatty acid
LFC: liver fat content
LDL: low-density lipoprotein
MR: magnetic resonance
MRI-PDFF: magnetic resonance imaging proton density fat fraction
MRS: magnetic resonance spectroscopy
NAFL: non-alcoholic fatty liver
NAFLD: non-alcoholic fatty liver disease
NASH: non-alcoholic steatohepatitis
NFS: NAFLD fibrosis score
OR: odds-ratio
SD: standard deviation
TyG: triglyceride-glucose
VLSFA: very long-chain saturated fatty acid
