# Supplementary material for: Plasma ceramides are associated with MRI-based liver fat content but not with noninvasive scores of liver fibrosis in patients with type 2 diabetes
Source: Cardiovasc Diabetol. 2023 Nov 8;22:310. doi: 10.1186/s12933-023-02049-2 (PMC10634084; doi:10.1186/s12933-023-02049-2)
Supplement: Supplementary file 1 — Supplementary Table 1. Quantified ceramide species and conditions for mass spectrometry acquisition. [file 12933_2023_2049_MOESM1_ESM.docx]

**Supplementary Table 1** Quantified ceramide species and conditions for mass spectrometry acquisition.

| **Molecular species** | **Mass transition (m/z)** | **CE**  **(eV)** | **Retention time**  **(min)** |
| --- | --- | --- | --- |
|  |  |  |  |
| d18:1/16:0 ceramide | 520.4⭢264.2 | 25 | 8.2 |
| d18:1/17:0 ceramide (IS) | 534.6⭢264.2 | 25 | 8.4 |
| d18:1/18:0 ceramide | 548.4⭢264.2 | 25 | 8.6 |
| d18:1/20:0 ceramide | 576.4⭢264.2 | 25 | 9.1 |
| d18:1/22:0 ceramide | 604.4⭢264.2 | 25 | 9.6 |
| d18:1/24:0 ceramide | 632.4⭢264.2 | 25 | 10.2 |
| d18:1/24:1 ceramide | 630.4⭢264.2 | 25 | 9.7 |
| d18:1/26:0 ceramide | 660.4⭢264.2 | 25 | 10.9 |

CE, collision energy; IS, internal standard.
