# Supplementary material for: Plasma ceramides are associated with MRI-based liver fat content but not with noninvasive scores of liver fibrosis in patients with type 2 diabetes
Source: Cardiovasc Diabetol. 2023 Nov 8;22:310. doi: 10.1186/s12933-023-02049-2 (PMC10634084; doi:10.1186/s12933-023-02049-2)
Supplement: Supplementary file 2 — Supplementary Table 2. Patient characteristics in LIRA-NAFLD. [file 12933_2023_2049_MOESM2_ESM.docx]

**Supplementary Table 2** Patient characteristics in LIRA-NAFLD

| **Characteristic** | **All** | **LFC ≤ 5.56%** | **LFC > 5.56%** |
| --- | --- | --- | --- |
| no. participants | 80 | 14 | 66 |
|  |  |  |  |
| Clinical characteristics |  |  |  |
| Age, years | 56.8 ± 11.0 | 55.3 ± 8.83 | 57.1 ± 11.3 |
| Gender, % female (n) | 43.8 (35) | 35.7 (5) | 45.5 (30) |
| Body mass index, kg/m^2^ | 36.5 ± 7.0 | 32.4 ± 4.5 | 37.1 ± 7.1 * |
| ≥30 kg/m^2^, % (n) | 80.0 (64) | 57.1 (8) | 84.8 (56) * |
| Diabetes duration, years | 10.0 ± 9.3 | 14.4 ± 11.7 | 9.3 ± 8.7 |
| Tobacco (current or former), % (n) | 53.8 (43) | 35.7 (5) | 57.6 (38) |
| Hypertension, % (n) | 80.0 (64) | 71.4 (10) | 81.8 (54) |
| Retinopathy, % (n) | 13.8 (11) | 28.6 (4) | 10.6 (7) |
| Diabetes medications |  |  |  |
| Insulin, % (n) | 30.0 (24) | 42.9 (6) | 27.3 (18) |
| Metformin, % (n) | 95.0 (76) | 78.6 (11) | 98.5 (65) * |
| Sulfonylureas, % (n) | 58.8 (47) | 57.1 (8) | 59.1 (39) |
| Lipid-lowering agents |  |  |  |
| Statins, % (n) | 42.5 (34) | 20.6 (7) | 40.9 (27) |
| Fibrates, % (n) | 10.0 (8) | 0.0 (0) | 12.1 (8) |
| Ezetimibe, % (n) | 10.0 (8) | 14.3 (2) | 9.1 (6) |
|  |  |  |  |
| Routine blood markers |  |  |  |
| HbA1c, % | 9.9 ± 2.1 | 9.5 ± 2.2 | 9.9 ± 2.1 |
| HbA1c, mmol/mol | 84 ± 18 | 81 ± 17 | 85 ± 18 |
| Fasting plasma glucose, mmol/L | 9.44 ± 3.45 | 7.96 ± 2.76 | 9.65 ± 3.50 |
| eGFR, mL/min/1.73m^2^ | 100 [87-106] | 100 [91-110] | 100 [82-106] |
| Total cholesterol, mmol/L | 4.50 ± 1.15 | 4.31 ± 0.96 | 4.53 ± 1.18 |
| LDL-cholesterol, mmol/L | 2.46 ± 0.86 | 2.35 ± 0.92 | 2.48 ± 0.86 |
| HDL-cholesterol, mmol/L | 1.06 ± 0.34 | 1.17 ± 0.39 | 1.05 ± 0.33 |
| Triglycerides, mmol/L | 1.83 [1.50-2.68] | 1.60 [1.45-2.04] | 1.85 [1.50-2.74] |
| TyG index | 5.15 ± 0.38 | 4.94 ± 0.28 | 5.18 ± 0.39 * |
| AST, IU/L | 23.0 [16.0-31.0] | 17.0 [12.0-23.0] | 25.0 [17.0-31.0] |
| ALT, IU/L | 39.0 [29.0-53.5] | 25.0 [20.0-36.0] | 41.0 [31.0-55.0] * |
| GGT, IU/L | 53.0 [31.3-100] | 25.0 [20.0-53.0] | 54.0 [36.5-102] ** |

Data are means ± SD (for normally-distributed variables), medians [IQR] (for non-normally distributed variables) or percentages, as appropriate.

Abbreviations: AST, aspartate aminotransferase; ALT, alanine aminotransferase; GGT, gamma-glutamyl transferase; eGFR, estimated glomerular filtration rate; GLP-1, glucagon-like peptide-1; HDL, high-density lipoprotein; LDL, low-density lipoprotein; TyG, triglyceride-glucose index.

LFC ≤ 5.56% vs. LFC > 5.56%: * p < 0.05, and ** p < 0.01.
