# Supplementary material for: Plasma ceramides are associated with MRI-based liver fat content but not with noninvasive scores of liver fibrosis in patients with type 2 diabetes
Source: Cardiovasc Diabetol. 2023 Nov 8;22:310. doi: 10.1186/s12933-023-02049-2 (PMC10634084; doi:10.1186/s12933-023-02049-2)
Supplement: Supplementary file 3 — Supplementary Table 3. Multivariate logistic regression analysis for liver fat content > 5.56%. [file 12933_2023_2049_MOESM3_ESM.docx]

**Supplementary Table 3** Multivariate logistic regression analysis for liver fat content > 5.56%.

|  | **GEPSAD** (n = 255) | | **LIRA-NAFLD** (n = 80) | |
| --- | --- | --- | --- | --- |
|  | Odds-ratio [95% CI] | P-value | Odds-ratio [95% CI] | P-value |
|  |  |  |  |  |
| **Total ceramide (log)** | **8.2 [1.6 - 46]** | **0.014** | **6.7 [1.2 - 38]** | **0.048** |
| 16:0 ceramide (log) | 0.02 [0.00 - 31.1] | 0.29 | 0.01 [0.00 - 27] | 0.73 |
| 18:0 ceramide (log) | 7x10^5^ [0.5 - 7x10^12^] | 0.07 | 9x10^5^ [1.2 - 9x10^12^] | **0.037** |
| 20:0 ceramide (log) | 3.51 [0.75 - 17.1] | 0.11 | 2.3 [0.24 - 9.2] | 0.48 |
| **22:0 ceramide (log)** | **3.9 [1.0 - 16]** | **0.05** | **4.1 [1.1 - 15]** | **0.031** |
| 24:1 ceramide (log) | 3.3 [0.8 - 15] | 0.11 | 1.5 [0.4 - 4.8] | 0.42 |
| **24:0 ceramide (log)** | **9.1 [2.1 - 43]** | **0.004** | **5.3 [1.9 - 36]** | **0.027** |
| 26:0 ceramide | 0.001 [0.00 - 75] | 0.24 | 0.001 [0.00 - 54] | 0.63 |
| VLSFA ceramides (log) | **8.9 [2.0 - 44]** | **0.005** | 3.6 [0.5 - 23] | 0.07 |
| LCFA ceramides | 0.51 [0.01 - 7.1] | 0.36 | 0.12 [0.01 - 4.1] | 0.65 |

The model is adjusted for age, diabetes duration, body mass index and dyslipidemia. Bolded: p < 0.05.

Abbreviations: CI, confidence intervals; LCFA, long-chain fatty acid; VLSFA, very long-chain saturated fatty acid.
