# Supplementary material for: Plasma ceramides are associated with MRI-based liver fat content but not with noninvasive scores of liver fibrosis in patients with type 2 diabetes
Source: Cardiovasc Diabetol. 2023 Nov 8;22:310. doi: 10.1186/s12933-023-02049-2 (PMC10634084; doi:10.1186/s12933-023-02049-2)
Supplement: Supplementary file 4 — Supplementary Table 4. Liver fat content and noninvasive scores of liver fibrosis in LIRA-NAFLD. [file 12933_2023_2049_MOESM4_ESM.docx]

**Supplementary Table 4** Liver fat content and noninvasive scores of liver fibrosis in LIRA-NAFLD

| **Characteristic** | **All** | **LFC ≤ 5.56%** | **LFC > 5.56%** |
| --- | --- | --- | --- |
| no. subjects | 80 | 14 | 66 |
|  |  |  |  |
| Liver fat content using MRI-PDFF | |  |  |
| Liver fat content (%) | 17.3 [8.07-27.6] | 3.31 [1.73-3.96] | 20.4 [11.5-30.8] *** |
| > 5.56%, % (n) | 82.5 (66) | 0.0 (0) | 100 (66) *** |
|  |  |  |  |
| Noninvasive scores of fibrosis | |  |  |
| FIB-4 | 1.005 [0.567-1.255] | 0.589 [0.524-1.561] | 1.026 [0.596-1.251] |
| < 1.30, % (n) | 78.8 (63) | 71.4 (10) | 80.3 (53) |
| ≥ 2.67, % (n) | 0.0 (0) | 0.0 (0) | 0.0 (0) |
| NFS | 0.019 ± 1.312 | -0.417 ± 1.355 | 0.089 ± 1.303 |
| < -1.455, % (n) | 15.0 (12) | 28.6 (4) | 12.1 (8) |
| ≥ 0.676, % (n) | 27.5 (22) | 21.4 (3) | 28.8 (19) |
| FNI | 0.546 ± 0.306 | 0.379 ± 0.374 | 0.570 ± 0.291 |
| < 0.10, % (n) | 12.5 (10) | 28.6 (4) | 9.1 (6) |
| ≥ 0.33, % (n) | 72.5 (58) | 28.6 (4) | 81.8 (54) *** |

Data are means ± SD (for normally-distributed variables), medians [IQR] (for non-normally distributed variables) or percentages, as appropriate.

Abbreviations: FIB-4, fibrosis-4 index; FNI, fibrotic NASH index; LFC, liver fat content; MRI-PDFF, magnetic resonance imaging proton density fat fraction; NFS, NAFLD fibrosis score.

LFC ≤ 5.56% vs. LFC > 5.56%: *** p < 0.001.
