# Supplementary figures and images for: Plasma ceramides are associated with MRI-based liver fat content but not with noninvasive scores of liver fibrosis in patients with type 2 diabetes
Source: Cardiovasc Diabetol. 2023 Nov 8;22:310. doi: 10.1186/s12933-023-02049-2 (PMC10634084; doi:10.1186/s12933-023-02049-2)

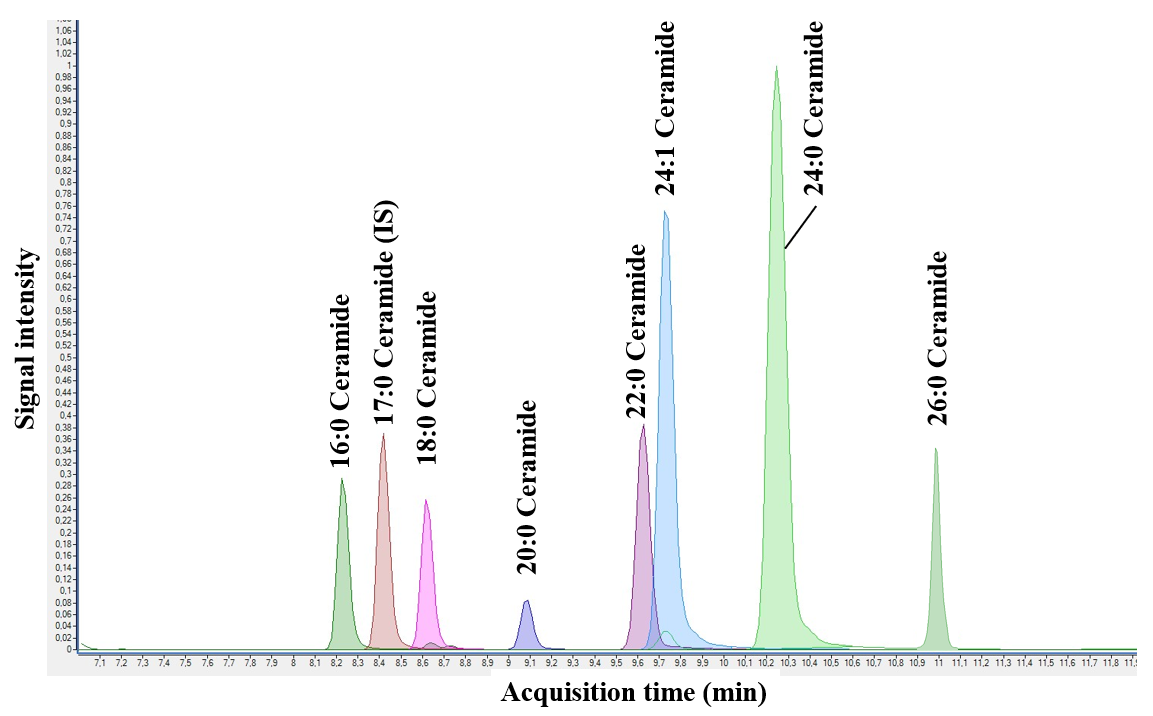


**Supplementary Fig. 1** A representative chromatogram of external and internal standards.

Supplement: Supplementary file 5 — Supplementary Fig. 1. A representative chromatogram of external and internal standards. [file 12933_2023_2049_MOESM5_ESM.docx]
